# Finite Element Analysis of Spinal Cord Stress in a Single Segment Cervical Spondylotic Myelopathy

**DOI:** 10.3389/fsurg.2022.849096

**Published:** 2022-03-14

**Authors:** Shaofeng Yang, Luqiang Qu, Lijie Yuan, Junjie Niu, Dawei Song, Huilin Yang, Jun Zou

**Affiliations:** ^1^Department of Orthopaedic Surgery, The First Affiliated Hospital of Soochow University, Suzhou, China; ^2^Department of Orthopaedic Surgery, Taicang Affiliated Hospital of Soochow University, Taicang, China

**Keywords:** cervical spondylotic myelopathy, finite element analysis, vertebral canal volume, maximum stress, spinal cord compression stress

## Abstract

**Background:**

Spinal cord ischemia is largely caused by cervical spondylotic myelopathy (CSM), which has a corresponding biomechanical basis. Finite element analysis of spinal cord stress in diseased segments of CSM was performed to provide a biomechanical basis for the pathogenesis of CSM.

**Methods:**

A single segment (C4-5) in a patient with CSM was selected for mechanical simulation of three-dimensional (3D) computed tomography scanning, and a 3D finite element model of the cervical vertebra was constructed. Based on the patient's age, sex, height, weight, and other parameters, a finite element analysis model of an individual with healthy cervical vertebrae in our hospital was selected as the control to compare the stress changes between the patient and control groups in the analysis of the cervical vertebrae under anterior flexion, posterior extension, lateral flexion, and rotating load in the diseased spinal cord segment.

**Results:**

In the CSM patient, the diseased segment was C4-5. Under loading conditions of forward flexion, posterior extension, left flexion, right flexion, left rotation, and right rotation, the maximum stress on the spinal cord in the control group was 0.0044, 0.0031, 0.00017, 0.00014, 0.0011, and 0.001 MPa, respectively, whereas those in the spinal cord in the CSM group were 0.039, 0.024, 0.02, 0.02, 0.0194, and 0.0196 MPa, respectively.

**Conclusion:**

The maximum stress on the diseased segments of the spinal cord in the CSM group was higher than that in the control group, which contributed to verifying the imaging parameters associated with spinal cord compression stress.

## Background

Cervical spondylotic myelopathy (CSM) is caused by spinal cord compression in the spinal canal due to degeneration of the cervical vertebrae, intervertebral discs, and ligaments (1, 2). Its incidence is high, and the early symptoms are often hidden. The symptoms appear when most cases progress to the middle and late stages, and irreversible damage to the spinal cord occurs

(3, 4). The pathophysiological mechanism of CSM mainly includes the following three aspects: anatomic abnormalities, kinetic factors, and spinal cord ischemia. Anatomical abnormalities and kinetic factors are mutually causal, and spinal cord ischemia is largely secondary to the above two factors, indicating that spinal cord injury caused by CSM has a corresponding biomechanical basis (5). In general, the methods of spinal biomechanics research mainly include experimental biomechanics and theoretical biomechanics. Experimental biomechanics mainly refers to the use of various models for biomechanical research, including experimental animals, cadaver specimens, and physical materials, but these models have certain limitations. Theoretical biomechanics refers to the biomechanical research carried out through theoretical calculations. With the development of computer science and technology, finite element calculations have been gradually applied widely in the biomechanical research of orthopedics. Recent studies (6–8) suggest that the fusion of three-dimensional (3D) finite element models and biomechanical models based on images can simulate the stress state of joints more accurately. Brekelmans et al. (9) used this method to preliminarily analyze the influence of vertebral body material properties and geometry on the stress distribution of intervertebral discs. In this study, a 3D finite element model of a single-segment CSM was constructed based on normal computed tomography (CT) scan images, and the stress changes of the diseased segments (C4-5) of the spinal cord under daily anterior flexion, posterior extension, lateral flexion, and rotating load were investigated to explore the pathogenesis of CSM.

## Methods

This study was approved by the Medical Ethics Committee of the First Affiliated Hospital of Soochow University. Selection of subjects: A female participant with typical symptoms of CSM (Japanese Orthopedic Association score of 10) was randomly selected from the clinically confirmed cases of CSM at the First Affiliated Hospital of Soochow University. After participant selection, the purpose of this study, implementation method, risks, and benefits were explained, and informed consent was obtained. Based on the age, sex, height, and weight of the patient, an existing healthy cervical spine finite element analysis model of our team was selected as the control.

### Establishment of Cervical (C4-5) Finite Element Model

To materialize two-dimensional CT data, DICOM files need to be converted and processed. At present, CT, magnetic resonance imaging (MRI), and other medical image workstations adopt volumetric 3D reconstruction, which cannot be directly used in engineering processing. Mimics software, developed by Materialize, Belgium, is a tool for the segmentation and processing of CT and MRI images. DICOM data were imported into Mimics software, and the view direction was set. The sagittal plane, coronal plane, and cross section were defined, and multiple DICOM data were sequentially discharged. At the interface, grayscale images, including bone tissue and background, were obtained. First, the image was preprocessed to improve its resolution and smoothness. Mimics software was used to perform regular treatment of the marrow cavity. According to the grayscale values of tissues of different densities, the corresponding thresholding interval was set by using the “Thresholding” command to extract the image data of bone-removing tissues. Using Boolean operation, models including the peri-osseous facet joints and intervertebral discs were obtained. At this point, there were many artifacts, holes, and noise in the model. The self-extraction function and erasure and filling function of the software were used to improve the quality of the tissue images layer by layer. Rough models of bone and soft tissue were obtained and saved in the STL file format. Geomagics exported the 3D model data in the STP format and imported it to PROE5.0 for model assembly and to manipulate the parts or features that needed to be processed.

The material properties (10) used in recent studies on the CSM are shown in Table 1. The ROM of the intact model at C4/5 was 7.56° in flexion, 6.21° in extension, 5.81° in lateral bending, and 4.51° in axial rotation (11). Based on the relaxed and analytical model of the cervical spine, the stress distribution diagrams of the patients with CSM and control were compared, and the stress change and maximum stress difference of the spinal cord at the C4-5 segments were compared between the two cervical vertebrae under anterior flexion, posterior extension, lateral flexion, and rotating load.

**Table 1:** Material properties of finite element analysis models.

**Component**	**Young modulus (MPa)**	**Poisson ratio**
Cortical bone	12,000	0.3
Cancellous bone	100	0.2
Bony end-plate	24	0.25
Pedicle	3,500	0.25
Small joints	15	0.45
Gray matter of spinal cord	0.656	0.499
ALL	20	0.3
PLL	70	0.3
LF	50	0.3
Soft backbone	142	0.45
Nucleus pulposus	1	0.499
Fiber ring	4.2	0.45
White matter of spinal cord	0.277	0.499

*ALL, anterior longitudinal ligament; PLL, posterior longitudinal ligament; LF, ligamentum flavum*.

## Results

**Figure 1:**
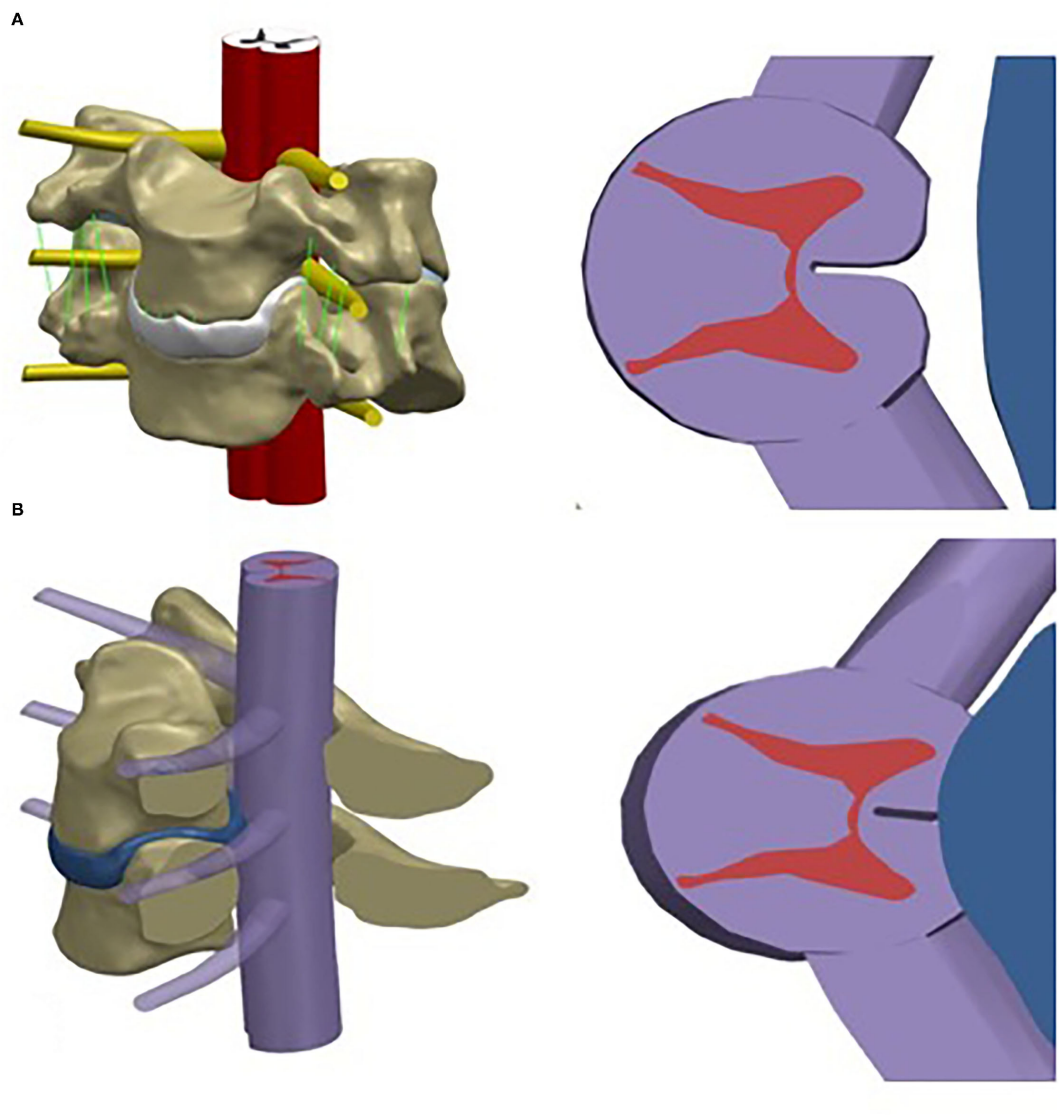
Assembly of the spinal cord geometric model and cervical finite element model (C4-5), and the transection of the geometric model of the spinal cord. **(A)** control subject; **(B)** CSM patient.

**Figure 2:**
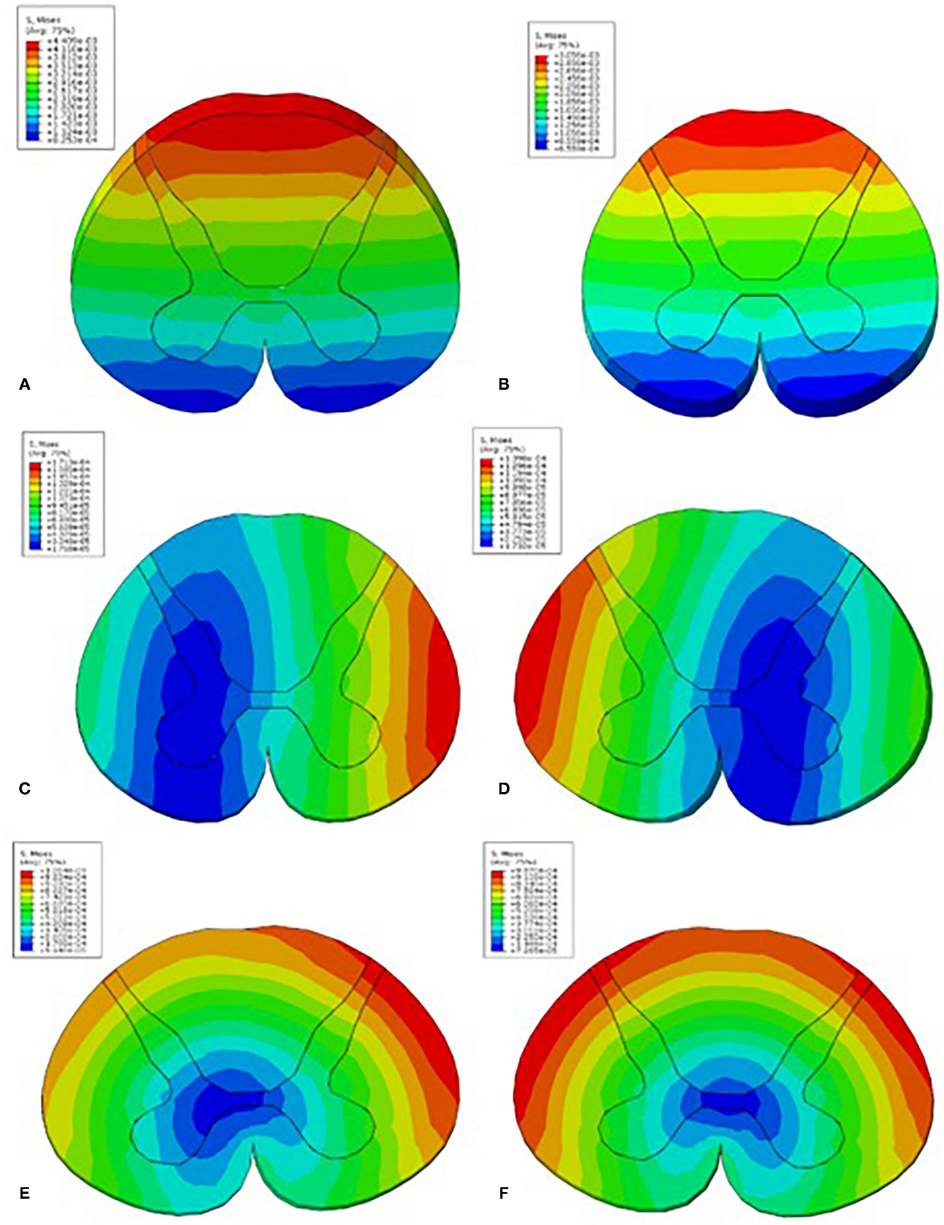
The Mises stress in the control patient under loading conditions in the C4-5 segment of forward flexion **(A)**, posterior extension **(B)**, left flexion **(C)**, right flexion **(D)**, left rotation **(E)**, and right rotation **(F)**.

**Figure 3:**
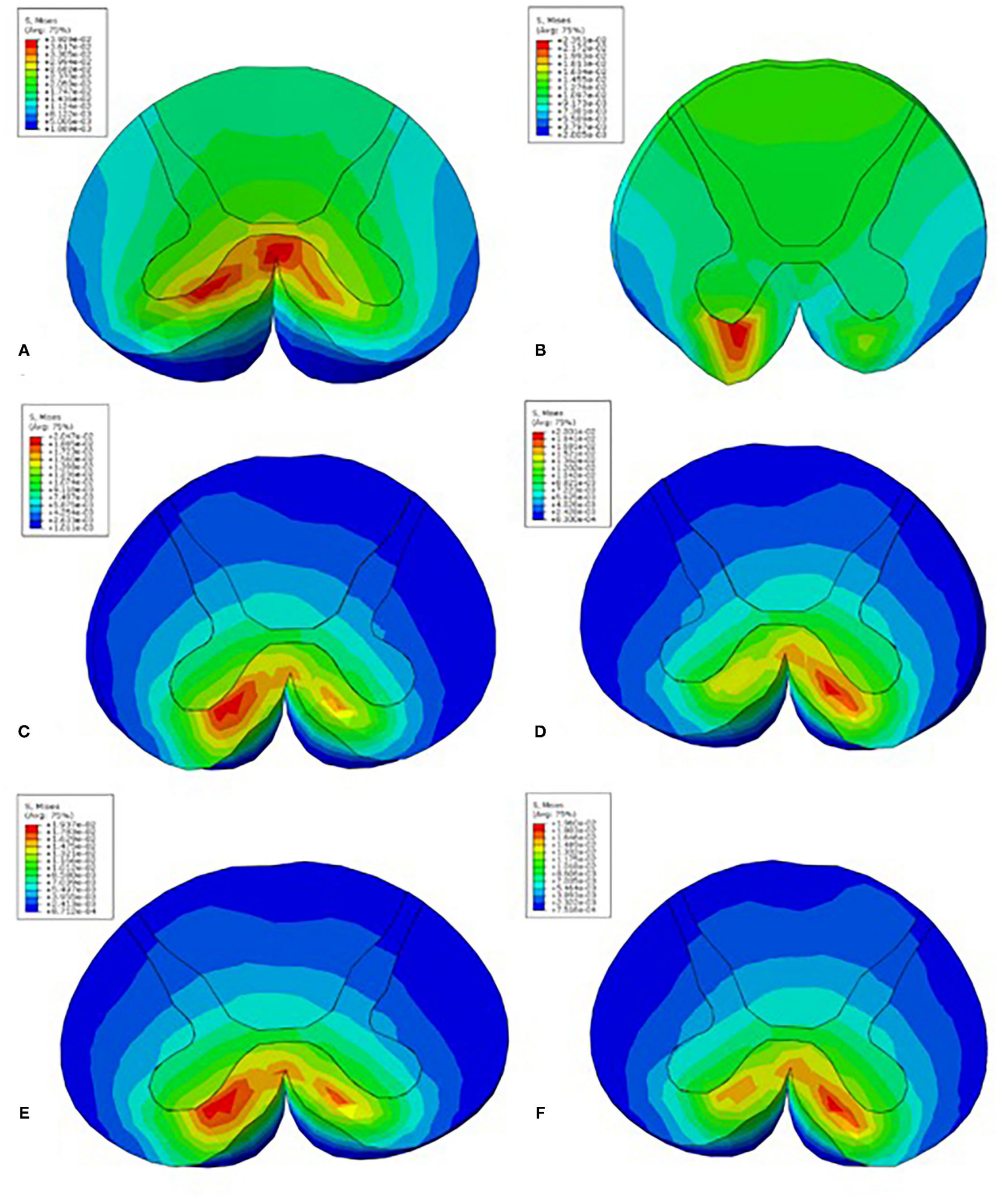
The Mises stress in the CSM patient under loading conditions in the C4-5 segment of forward flexion **(A)**, posterior extension **(B)**, left flexion **(C)**, right flexion **(D)**, left rotation **(E)**, and right rotation **(F)**.

The C4-5 finite element models of the control and patients with CSM are shown in Figures 1A,B, respectively. The von Mises stress in the control and CSM patients was extracted from the spinal cord at the corresponding position of the disc under the corresponding load, and the stress distribution nephograms are shown in Figures 2, 3. Under the loading conditions of forward flexion (Figure 2A), posterior extension (Figure 2B), left flexion (Figure 2C), right flexion (Figure 2D), left rotation (Figure 2E), and right rotation (Figure 2F), the maximum stress of the spinal cord in the control group was 0.0044, 0.0031, 0.00017, 0.00014, 0.0011, and 0.001 MPa, respectively, while the maximum stress of the spinal cord in the CSM group was 0.039 (Figure 3A), 0.024 (Figure 3B), 0.02 (Figure 3C), 0.02 (Figure 3D), 0.0194 (Figure 3E), and 0.0196 MPa (Figure 3F), respectively.

## Discussion

CSM is a complex disease caused by a combination of factors, including congenital spinal stenosis, static compression of the spinal cord due to degenerative changes, and dynamic impingement secondary to micromotion of the spinal column. It represents the most common cause of dysfunction in people over the age of 55 (12) and is found in 10–15% of all patients with cervical spondylosis. It can present in many ways and is typically characterized by neurological dysfunction, matching the pattern of spinal cord compression seen on radiography. Modern imaging modalities, especially MRI, with excellent soft tissue contrast, greatly facilitate the diagnosis and surgical planning of CSM (13). However, it generally provides a neutral supine evaluation and does not account for dynamic pathophysiological factors that may be present only during postural extension (14, 15).

In general, the methods of spinal biomechanics research mainly include experimental biomechanics and theoretical biomechanics. Experimental biomechanics mainly refers to the use of various models for biomechanical research, including experimental animals, cadaver specimens, and physical materials, but these models have certain limitations. Theoretical biomechanics refers to the biomechanical research carried out through theoretical calculations. With the development of computer science and technology, finite element calculations represented by it have been gradually applied widely in biomechanical research of orthopedics, especially the spine, as a supplement to clinical research and cadaver experimental models *in vitro* (16). In biomechanical evaluation, the load modes most commonly used and closest to physiological motion are generally applied in flexion, extension, lateral flexion, and rotation. Therefore, the experimental results of the model are often compared with those of previous 3D finite element models. After modeling, a certain amount of torque value is usually applied to the model for pre-loading, and the stress of the model under loading conditions such as forward bending, backward extension, lateral bending, and rotation is observed.

The cervical biomechanical behavior follows the non-linear distribution of each component; hence, previous studies (17, 18) focused on computer modeling and analysis of discs. This study focused on the vertebral body, intervertebral discs, ligaments, and spinal cord during cervical spine non-linear stress conditions and the overall analysis of the cervical spine and changes in soft tissue morphology of the neck in patients with CSM and control. The stress condition after the retroflexion movement was stereologically reproduced. In this study, the control and CSM patients were subjected to the same external force. Due to the normal structure and function of the healthy cervical vertebra, the curvature of the cervical vertebra increases when an external force is applied, that is, the cervical vertebra is displaced. At this point, the cervical vertebrae of the control subjects in our study could withstand greater stress without injury. However, due to changes in the physiological curvature of the cervical vertebra in patients with CSM, the mechanics of the main components of the cervical vertebrae were unbalanced, the stress that the neck could bear was reduced, and the range of motion of the cervical vertebra was reduced.

This study had some limitations. Although special attention and analysis are given during model development, finite element analysis has limitations, similar to cadaver studies and other published finite element studies. Simple elastic model for analysis in this study. Other hyperelastic or hyperporoelastic models can be considered in future studies. Caution should be used in interpreting the results of this study, as the complete finite element analysis was based on a single scan of a normal male. The purpose of computational simulations is to provide trends rather than actual data. Comparisons in the finite element analysis were not statistically significant. It is just a biomechanical trend analysis and comparison, similar to many finite element analysis studies. In our finite element study, the neck muscles were missing. Muscles mainly control the range of motion of the cervical spine. The loss of neck muscles may have an impact on the biomechanical values of the limited units. In addition, the von Mises stress is a simple stress parameter, which has some limitations.

## Conclusion

The maximum stress on the diseased segments of the spinal cord in the CSM group was higher than that in the control group, which verified the above imaging parameters associated with spinal cord compression stress.

## Data Availability Statement

The raw data supporting the conclusions of this article will be made available by the authors, without undue reservation.

## Ethics Statement

This clinical study was approved by the Ethics Committee of the First Affiliated Hospital of Soochow University, and written informed consent was obtained from all participants.

## Author Contributions

SY and LQ were responsible for designing and drafting the manuscript. LY, JN, and DS performed the data collection and statistical analyses. HY and JZ critically revised the manuscript. All authors read and approved the final manuscript.

## Conflict of Interest

The authors declare that the research was conducted in the absence of any commercial or financial relationships that could be construed as a potential conflict of interest.

## Publisher's Note

All claims expressed in this article are solely those of the authors and do not necessarily represent those of their affiliated organizations, or those of the publisher, the editors and the reviewers. Any product that may be evaluated in this article, or claim that may be made by its manufacturer, is not guaranteed or endorsed by the publisher.

## Abbreviations

CSM: cervical spondylotic myelopathy
CT: computed tomography
MRI: magnetic resonance imaging.
